# Supplementary material for: The causal relationship between the human gut microbiota and pyogenic arthritis: a Mendelian randomization study
Source: Front Cell Infect Microbiol. 2024 Nov 26;14:1452480. doi: 10.3389/fcimb.2024.1452480 (PMC11629706; doi:10.3389/fcimb.2024.1452480)
Supplement: Supplementary file 1 [file DataSheet1.zip › mendelian test/ebi-a-GCST90027497.csv_forest.pdf]

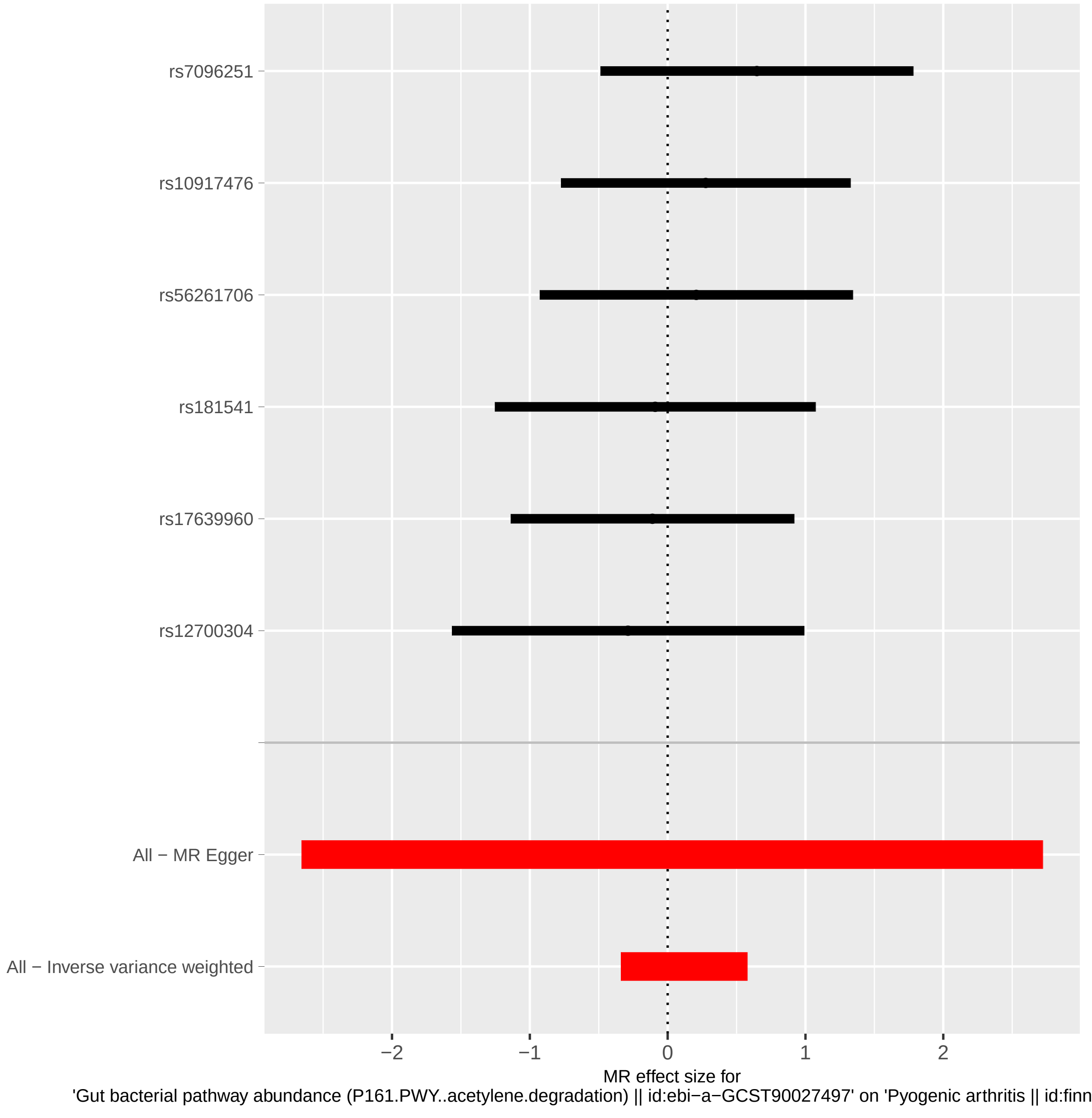
rs7096251
rs10917476
rs56261706
rs181541
rs17639960
rs12700304
All − MR Egger
All − Inverse variance weighted
−2
−1
0
1
2
MR effect size for
'Gut bacterial pathway abundance (P161.PWY..acetylene.degradation) || id:ebi−a−GCST90027497' on 'Pyogenic arthritis || id:finn
